# Age-related biomechanical variations in vertical jumping and sprinting performance among basketball players

**DOI:** 10.3389/fspor.2024.1488128

**Published:** 2025-01-15

**Authors:** Milos Petrovic, Jorgelina Ramos, Thrainn Hafsteinsson, Thordis Gisladottir

**Affiliations:** ^1^ Research Centre for Sports and Healthcare Sciences, University of Iceland, Reykjavik, Iceland; ^2^ Department of Endocrinology, Landspitalin, Reykjavik, Iceland

**Keywords:** basketball, biomechanics, force-time analysis, countermovement jump, sprinting

## Abstract

**Introduction:**

This study aims to investigate age-related differences in physical performance metrics, specifically vertical jumping and sprinting capabilities, between young (average age 12.5) and senior (average age 23.2) male basketball players.

**Methods:**

Performance metrics were assessed through standardised tests measuring jump height and sprint times, alongside force production during eccentric and concentric phases of jumping movement.

**Results:**

Key findings show that senior players outperform younger athletes in both sprint times and jump heights, attributed to greater physical maturation and neuromuscular development. However, contraction times and phase durations were similar across both age groups, indicating that strength improvements do not affect fundamental movement timing.

**Discussion:**

These results highlight the importance of age-specific training programs that focus on developing explosive power for younger players and optimising performance while minimising injury risk for seniors. The study provides valuable insights and recommendations for tailoring training strategies to athletes' developmental stages and suggests that further research is needed to explore effective interventions for enhancing physical performance across the lifespan.

## 1 Introduction

The physical demands of basketball require an understanding of key performance metrics, such as vertical jumping and sprinting, which are critical for success in the sport. These metrics reflect an athlete's explosive power, speed, and overall athleticism (1). As athletes age, these attributes evolve due to physiological, anatomical, and neuromuscular changes (2). Understanding these changes is crucial for optimising training programs and ensuring peak performance at different stages of athletic development.

Vertical jumping ability is fundamental in basketball, influencing actions such as shooting, rebounding, and shot blocking (3). Jumping mechanics vary between younger and older athletes, influenced by muscle mass, tendon stiffness, and neuromuscular coordination (4, 5). As athletes age, reductions in these factors can lead to decreases in jump height and efficiency, along with slower reaction times and diminished explosive power. These declines not only affect performance but also increase the risk of injury, as the body's ability to generate and absorb force diminishes (6, 7). Understanding the age-related changes in jumping mechanics is essential for designing interventions that help maintain or improve performance while reducing injury risk in older players (8).

Similarly, sprinting performance, vital for fast breaks and defensive recovery, also changes with age. Sprinting efficiency is influenced by factors such as stride length, ground contact time, and muscle fibre composition, all of which develop differently across age groups (9, 10). Older athletes may experience declines in sprinting speed due to a decrease in fast-twitch muscle fibres and alterations in joint mechanics. Age-related changes in muscle elasticity and joint range of motion can also impair energy transfer during sprinting, leading to slower acceleration and deceleration (11, 12). These shifts reduce an athlete's ability to maintain high-intensity efforts, affecting their overall performance on the court. Understanding these changes is crucial for developing training programs that address the specific needs of athletes at different stages of their careers (13).

This study compares young and senior male basketball players to examine the biomechanical evolution that occurs with age. By focusing on vertical jumping and sprinting, we aim to identify age-related differences in performance. Our hypothesis is that these differences, particularly in mechanics and efficiency, will highlight the need for age-specific training programs to maximise performance and minimise injury risks throughout an athlete's career.

## 2 Materials and methods

### 2.1 Participants

Twenty male basketball players (age: 12.5 ± 1.2 years; body height: 160 ± 9.9 cm, body weight: 53.8 ± 12.5 kg) and thirteen male senior basketball players (age: 23.2 ± 3.9 years; body height: 192 ± 8.6 cm, body weight: 88.6 ± 13 kg) volunteered for this study. All were active members of their basketball teams and free from musculoskeletal injuries that could affect the testing procedures and their performance. Participants suffering a lower limb injury within 6 months prior to the tests were excluded from the study. The testing procedures were approved by the University of Iceland Ethical Board (SHV2023-048) and parental consent was obtained for each underage participant. Senior players signed the consent forms on their own.

Participants in the younger group have attended three basketball sessions per week for the last 18 months, while senior members were attending 5–6 sessions per week, depending on the training and competition load.

### 2.2 Procedures

Upon arrival at the gym, the athletes completed a standardised warm-up administered by their strength and conditioning coach, which included dynamic stretching exercises such as forward/backpedal jog, forward/side lunges, high knees, butt kicks, carioca, and straight leg kicks. After becoming familiar with the testing procedures, each athlete stepped onto a uni-axial force plate system (ForceDecks Max, VALD Performance, Brisbane, Australia) and performed three maximal-effort countermovement jumps (CMJ) with their hands on their hips throughout the movement. The force plate system, sampling at 1,000 Hz, was calibrated/zeroed between each participant. To minimise fatigue, each jump trial was separated by approximately 15 s of rest. The average value of the three jumps was used for performance analysis. Verbal encouragement was provided to motivate participants to exert maximal effort and push off the ground as forcefully as possible.

For the linear sprint speed test, two photocells (SmartSpeed Plus timing gate system, VALD Performance, Brisbane, Australia) were placed at the start and at the 20-m marker. Participants began the sprint from a stationary standing start, with their preferred foot positioning on a marked line on the floor. Line was placed 1 m behind the photocells. The time was recorded from when they passed the first photocells until they crossed the 20-m finish line as quickly as possible. Each participant completed three trials, with a 3–4-min rest period between trials. The average time of the three trials was calculated for further analysis.

### 2.3 Variables

During the eccentric phase of the CMJ, the following variables were analysed: braking phase duration, eccentric duration, peak velocity, mean force, peak force, peak force/body mass (BM), mean power, mean power/BM. During the concentric phase of the CMJ, the variables examined included concentric duration, peak velocity, mean force, mean force/BM, peak force, peak force/BM. Additionally, contraction time, jump height, and modified reactive strength index (RSI-modified) were obtained. Jump height was calculated using the impulse-momentum relationship, while RSI-modified was calculated by dividing jump height by contraction time. Contraction time was defined as starting when the athlete's system mass was reduced by 20 N (movement onset) and ending at take-off. Take-off was marked when vertical force dropped below a threshold of 20 N. Following manufacturer recommendations, the eccentric phase was identified as the phase with negative centre of mass velocity. The braking phase, a sub-phase of the eccentric phase, started at minimum force and ended at the completion of the eccentric phase. Impulse within each sub-phase was calculated as the area under the force-time curve. Further details on CMJ force-time metrics are available in the VALD user manual (https://valdperformance.com/news/understanding-the-countermovement-jump). Running time in 20 m sprint were taken into further analysis.

### 2.4 Statistical analysis

Independent *t*-tests examined between-group statistically significant differences for normally distributed variables (mean and standard deviation). Statistical significance was set *a priori* at *p* < 0.05. All statistical analyses were conducted using Jamovi software (Jamovi Project, Version 2.3).

## 3 Results

The analysis revealed significant differences between younger and senior basketball players across several physical and performance metrics (Table 1). As expected, senior players were significantly older, taller, and heavier than their younger counterparts. They also demonstrated superior speed, with faster 20-m sprint times, and greater explosive power, as evidenced by higher jump heights.

**Table 1:** Descriptive data and comparison statistics, between younger and older basketball players for each dependent variable examined in the present study presented as a mean (standard deviation).

Independent samples *t*-test	Youngers	Seniors	*p*
Age [year]	12.5 (1.19)	23.2 (3.94)*	<.001
Body height [cm]	160 (9.85)	192 (8.16)*	<.001
Body weight [kg]	53.8 (12.5)	192 (8.16)*	<.001
20 m sprint [sec]	3.82 (0.32)	2.91 (0.08)*	<.001
Jump height (Imp-Mom) [cm]	27.5 (9.22)	40.3 (5.02)*	<.001
RSI-modified (Imp-Mom) [m/s]	0.42 (0.11)	0.53 (0.10)*	0.007
RSI-modified [m/s]	0.399 (0.17)	0.51 (0.01)*	0.039
Contraction time [ms]	747 (143)	808 (122)	0.22
Braking phase duration [s]	0.292 (0.07)	0.327 (0.06)	0.188
Eccentric duration [ms]	472 (140)	542 (94.6)	0.123
Eccentric peak velocity [m/s]	0.972 (0.354)	1.27 (0.354)*	0.014
Eccentric peak force [N]	1,037 (303)	2,080 (266)*	<.001
Eccentric peak force/BM [N/kg]	19 (2.97)	23.7 (2.89)*	<.001
Eccentric mean force [N]	537 (145)	871 (127)*	<.001
Eccentric mean power [W]	267 (120)	555 (98.2)*	<.001
Eccentric mean power/BM [W/kg]	4.8 (1.54)	6.34 (1.21)*	0.005
Concentric duration [ms]	275 (66.4)	266 (32.8)	0.638
Concentric peak velocity [m/s]	2.38 (0.4)	2.91 (0.16)*	<.001
Concentric peak force [N]	1,251 (356)	2,192 (341)*	<.001
Concentric peak force/BM [N/kg]	23 (2.29)	24.8 (1.84)*	0.025
Concentric mean force [N]	987 (267)	1,809 (270)*	<.001
Concentric mean force/BM [N/kg]	18.2 (2.04)	20.4 (1.15)*	0.001

Asterisk (*) means significantly different than youngers.

In terms of reactive strength, seniors outperformed the younger group in RSI-modified measurements. Force production during both eccentric and concentric phases was notably higher in senior players, highlighting their enhanced strength and power. However, no significant differences were found in contraction time, braking phase duration, or the duration of eccentric and concentric phases, indicating that while senior players are stronger and faster, the time characteristics of their movements were similar across groups.

Overall, the results indicate that senior players have developed superior physical attributes and performance capabilities compared to younger players, likely due to physical maturation and experience (13).

## 4 Discussion

The results of this study reveal significant age-related differences in physical and performance metrics between younger and senior basketball players, aligning with existing literature on athletic development. Senior players were significantly taller, heavier, and faster, and demonstrated superior performance in both sprinting and vertical jumping tasks. These findings are consistent with previous research that highlights the impact of physical maturation on athletic performance, particularly in sports requiring explosive power and speed (2).

The superior 20-m sprint times observed in senior players reflect their advanced development of fast-twitch muscle fibres, which are crucial for high-speed movements and quick accelerations (9, 10). This increased sprinting efficiency among senior athletes is likely due to a combination of greater muscle mass, enhanced neuromuscular coordination, and optimised biomechanical factors such as stride length and ground contact time (11). Moreover, the findings that senior players achieved higher jump heights, and better RSI-modified scores indicate that they possess superior reactive strength and explosive power, which are essential for high-intensity actions like rebounding and shot-blocking (3, 8). Gervasi et al. (14) suggest that after reaching the age of 17–18, the variations in RSI appear to plateau, meaning that significant gains are not observed beyond this developmental age. This plateau suggests that by this age, athletes have reached a level of maturation where further increases in RSI are less pronounced, possibly due to the completion of physiological development. The findings indicate that maturation plays a key role in jump performance, with the most significant improvements occurring during the early to mid-adolescent years.

An important consideration in interpreting these results is the emphasis on analysing force and power parameters relative to body weight, rather than relying solely on absolute values. The significant improvements in relative force production during both eccentric and concentric phases among senior players highlight their superior ability to generate power in proportion to their body mass. This is particularly relevant in basketball, where athletes must efficiently propel their own body weight during actions like jumping and sprinting (13, 15). By examining these parameters relative to body weight, we can gain a more accurate understanding of an athlete's true functional capabilities and how they translate to on-court performance.

Interestingly, while senior players exhibited significantly higher force production during both the eccentric and concentric phases of movement, the time characteristics of these movements—such as contraction time and phase durations—were similar across both groups. This suggests that while senior players have developed greater strength and power, the fundamental timing of their muscle contractions remains consistent with younger athletes. These findings highlight the importance of focusing not only on developing strength and power but also on maintaining or improving the efficiency of movement as athletes age (15).

The significant differences in eccentric and concentric force production, particularly when considered relative to body mass, underscore the advanced neuromuscular adaptations that occur with age and training experience. Senior players' ability to generate higher forces, both relative to body mass and in absolute terms, is likely a result of long-term training adaptations, increased muscle mass, and better coordination of motor units (4, 13). This enhanced force production capability is critical for basketball performance, where repeated high-intensity actions are required throughout a game.

In summary, the findings from this study support the hypothesis that age-related differences in physical and performance metrics exist between younger and senior basketball players. These differences are primarily driven by the physiological and biomechanical developments that occur with maturation and experience. The results underscore the importance of age-specific training programs that can address the unique needs of athletes at different stages of their careers, optimising their performance while minimising injury risks (7). Future research should further explore the specific training interventions that can help maintain or even enhance these physical attributes as athletes age. Additionally, incorporating a focus on relative force and power metrics can provide more nuanced insights into an athlete's functional capabilities, ensuring that training regimens are appropriately tailored to individual needs and body compositions. The observed differences highlight the impact of age and experience on performance, suggesting that targeted training programs could benefit younger players by focusing on strength and explosive power development. Future research could delve deeper into the mechanisms underlying these differences, such as neuromuscular adaptations and skill refinement. Studies by Haff et al. (16) and Stone et al. (17) provide a foundation for exploring how specific training regimens impact performance variables and could guide the development of more effective training strategies.

### 4.1 Practical guidelines for coaches and trainers

#### 4.1.1 Age-specific training

Younger Players (12–14 years): Focus on developing explosive power, neuromuscular coordination, and foundational strength through plyometrics, sprint drills, and bodyweight exercises. Emphasise injury prevention with proper warm-up and cool-down routines.

Senior Players (18 + years): Optimise performance with advanced resistance training, explosive power development, and sprint training to maintain strength, speed, and efficiency. Prioritise recovery strategies, including active recovery and mobility work.

### 4.2 Strength and power development

Incorporate dynamic strength training targeting both eccentric and concentric phases of movement. For both age groups, include reactive strength and plyometric drills to improve jump height and sprinting capabilities.

### 4.3 Sprint training

Younger Players: Focus on acceleration and start mechanics with resisted sprints and interval training.

Senior Players: Emphasise maximal velocity and speed endurance through longer sprints and sprint technique drills.

### 4.4 Sexual maturation considerations

Monitor individual maturation stages and tailor training to the player's physical development, using growth markers to adjust intensity and volume.

### 4.5 Performance monitoring

Regularly track vertical jump and sprint performance using tools like force plates and timing gates, providing feedback to motivate and guide progress. More focus has to be put on better understanding of RSI-mod and its variations during developmental stage (14).

We would like to acknowledge several important limitations of this study. While our aim was to explore the differences between younger and older male basketball players, further research is needed to consider the role of sexual maturation in these dynamics. Additionally, more studies are required to examine these factors in female basketball players. Although we compared different age groups, this study is a cross-sectional analysis, focusing on two distinct age cohorts at a single point in time. A longitudinal study tracking the younger players as they progress into their senior years would offer valuable insights into how time-force metrics evolve with maturation. Furthermore, we recognise that the sample size in this study is small and homogenous, and as such, we advise caution in generalising these findings.

In conclusion, the study supports the notion that age and experience play crucial roles in enhancing basketball performance. Senior players demonstrate superior physical attributes and performance capabilities compared to younger players, reflecting the benefits of physical maturation and extensive training. Further research into the interactions between these factors and performance variables will be valuable for optimising training programs and improving athletic outcomes across different age groups.

## Data Availability

The raw data supporting the conclusions of this article will be made available by the authors, without undue reservation.
